# Supplementary material for: A Novel Strategy to Identify Endolysins with Lytic Activity against Methicillin-Resistant Staphylococcus aureus
Source: Int J Mol Sci. 2023 Mar 17;24(6):5772. doi: 10.3390/ijms24065772 (PMC10059956; doi:10.3390/ijms24065772)
Supplement: Supplementary file 1 [file ijms-24-05772-s001.zip › 230301_LyJH1892_S_aureus_HKIM_Supplemental_Figure.pdf]

(a) Multi-alignment of CHAP domain

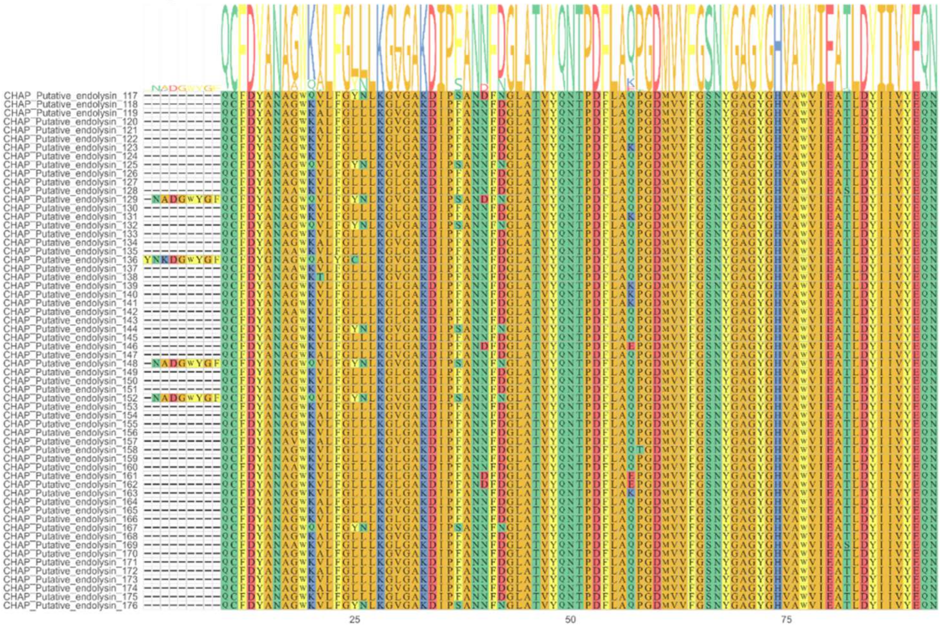

(b) Multi-alignment of Ami_2 domain

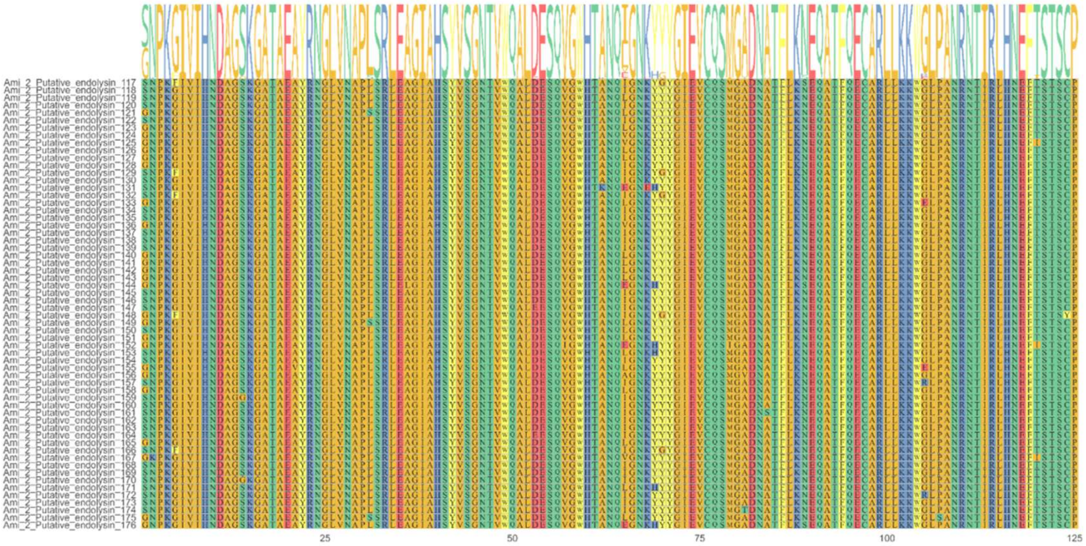

(c) Multi-alignment of SH3_5 domain

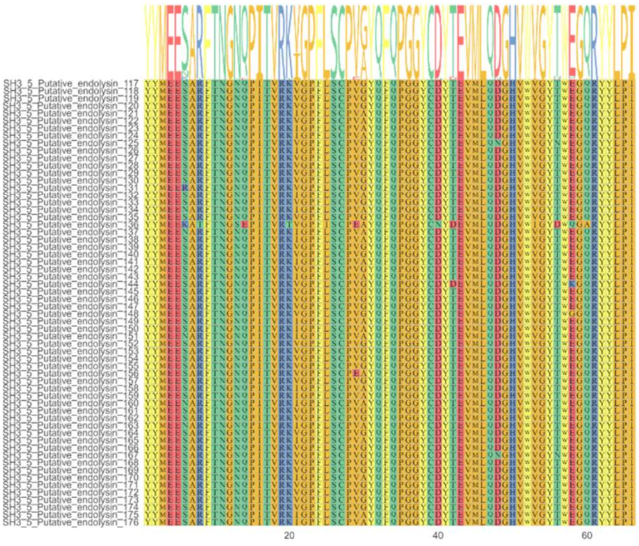

Figure S1. Multi-alignment of putative endolysins belonging to group 1 based on amino acids. (a) Multi-alignment of the cysteine, histidine-dependent amidohydrolases/peptidases domain (Accession: pfam05257 and sequence identity: 83.5%). (b) Multi-alignment of the Ami_2 domain (Accession: smart00644 and sequence identity: 82.4%). (c) Multi-alignment of the SH3_5 domain (Accession: pfam08460 and sequence identity: 75.8%).

(a) Multi-alignment of Ami_3 domain

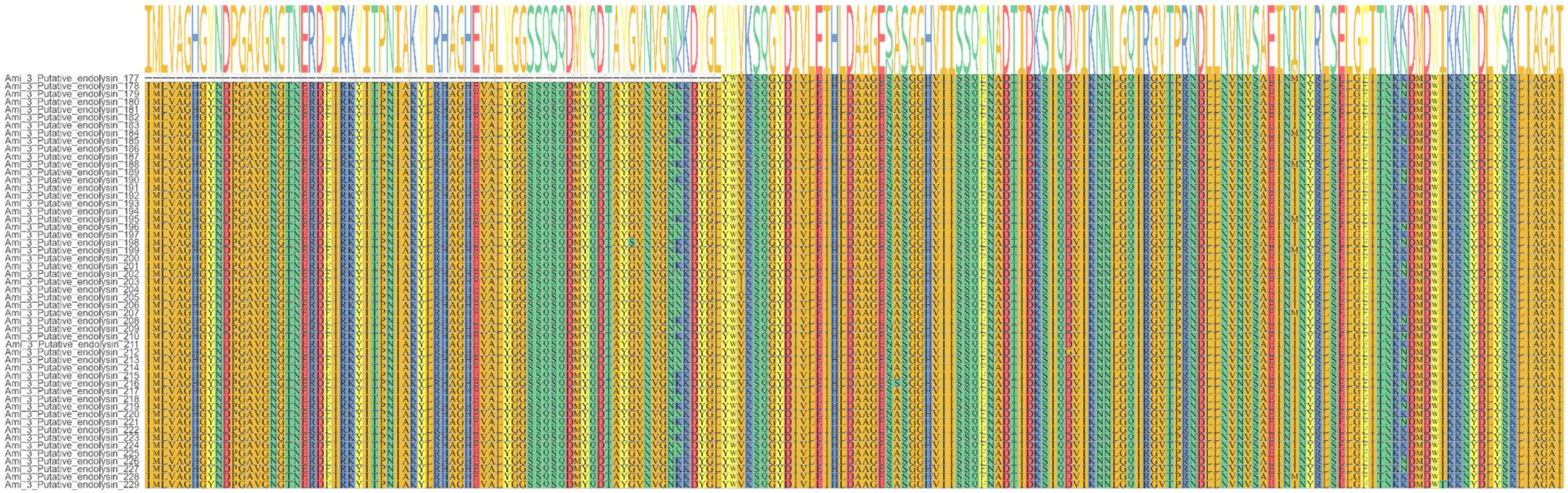

(b) Multi-alignment of SH3b domain

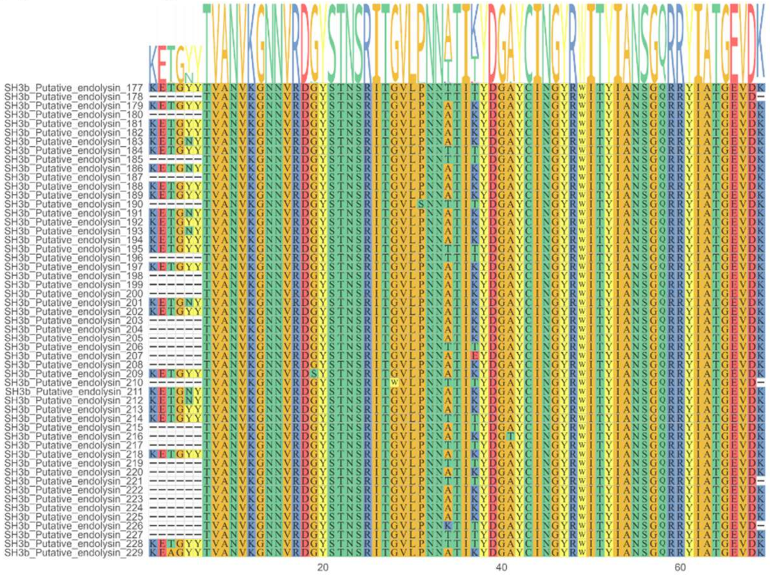

Figure S2. Multi-alignment of putative endolysins belonging to group 3 based on amino acids. (a) Multi-alignment of the Ami_3 domain (Accession: pfam01520 and sequence identity: 95.1%). (b) Multi-alignment of the SH3_b domain (Accession: smart00287 and sequence identity: 88.4%).

(a) NCCP 16830

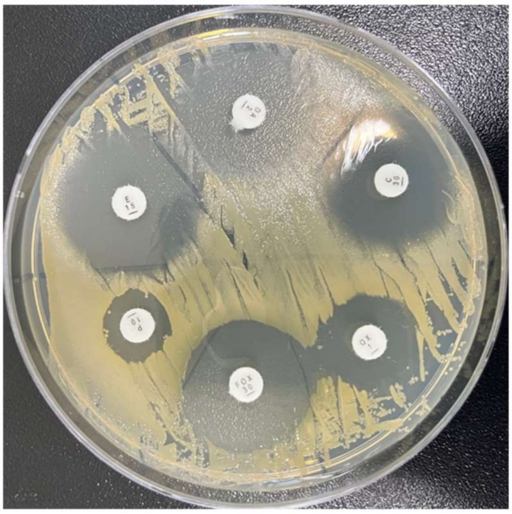

(b) NCCP 14567

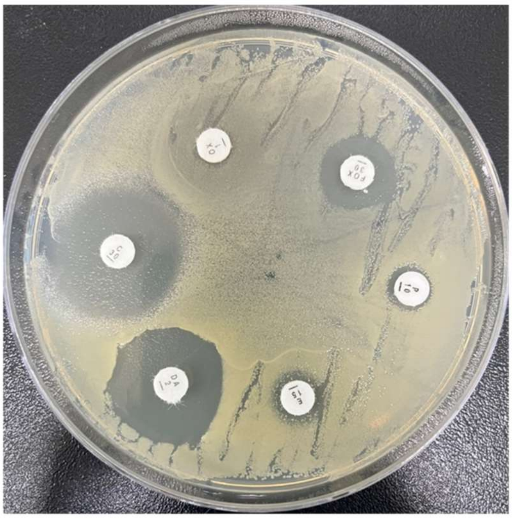

(c) NCCP 14754

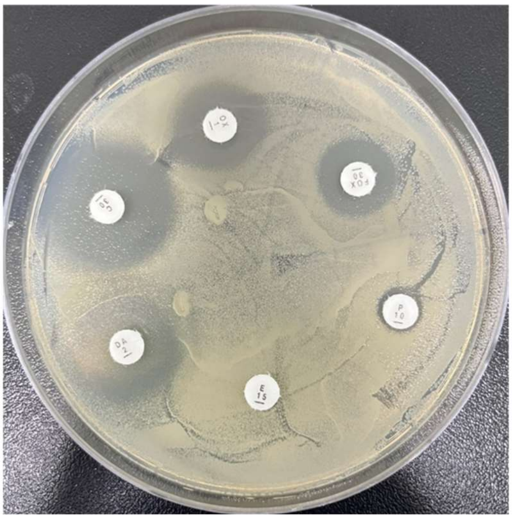

(d) KVCC-BA0500624

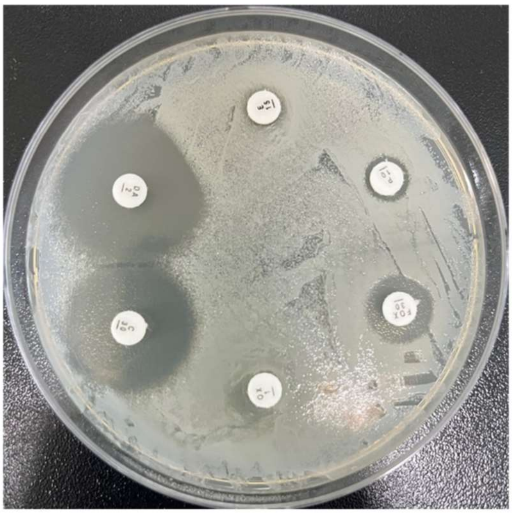

Figure S3. Antibiotic susceptibility test of *Staphylococcus aureus* strains. Cells of each *S. aureus* strain were spread on Mueller-Hinton agar, and antibiotic discs were placed on the plates. FOX, cefoxitin, 30 µg; OX, oxacillin, 1 µg; P, penicillin, 10 µg; E, erythromycin, 15 µg; DA, clindamycin, 2 µg; Chloramphenicol, 30 µg.
